# An Emerging Role for Epigenetic Dysregulation in Arsenic Toxicity and Carcinogenesis

**DOI:** 10.1289/ehp.1002114

**Published:** 2010-08-02

**Authors:** Xuefeng Ren, Cliona M. McHale, Christine F. Skibola, Allan H. Smith, Martyn T. Smith, Luoping Zhang

**Affiliations:** 1 Division of Environmental Health Sciences and; 2 Arsenic Health Effects Research Program, School of Public Health, University of California–Berkeley, Berkeley, California, USA

**Keywords:** arsenic carcinogenesis, arsenical compounds, DNA methylation, epigenetics, histone modification, microRNA

## Abstract

**Background:**

Exposure to arsenic, an established human carcinogen, through consumption of highly contaminated drinking water is a worldwide public health concern. Several mechanisms by which arsenical compounds induce tumorigenesis have been proposed, including oxidative stress, genotoxic damage, and chromosomal abnormalities. Recent studies have suggested that epigenetic mechanisms may also mediate toxicity and carcinogenicity resulting from arsenic exposure.

**Objective:**

We examined the evidence supporting the roles of the three major epigenetic mechanisms—DNA methylation, histone modification, and microRNA (miRNA) expression—in arsenic toxicity and, in particular, carcinogenicity. We also investigated future research directions necessary to clarify epigenetic and other mechanisms in humans.

**Data sources and synthesis:**

We conducted a PubMed search of arsenic exposure and epigenetic modification through April 2010 and summarized the *in vitro* and *in vivo* research findings, from both our group and others, on arsenic-associated epigenetic alteration and its potential role in toxicity and carcinogenicity.

**Conclusions:**

Arsenic exposure has been shown to alter methylation levels of both global DNA and gene promoters; histone acetylation, methylation, and phosphorylation; and miRNA expression, in studies analyzing mainly a limited number of epigenetic end points. Systematic epigenomic studies in human populations exposed to arsenic or in patients with arsenic-associated cancer have not yet been performed. Such studies would help to elucidate the relationship between arsenic exposure, epigenetic dysregulation, and carcinogenesis and are becoming feasible because of recent technological advancements.

The International Agency for Research on Cancer (IARC) classified arsenic, a toxic metalloid, as a group 1 carcinogen > 20 years ago (IARC 1987). It is widely accepted that exposure to arsenic is associated with lung, bladder, kidney, liver, and nonmelanoma skin cancers (IARC 2004; Pershagen 1981; Smith et al. 1992; Smith and Steinmaus 2009). High levels of arsenic have also been associated with the development of several other diseases and deleterious health effects in humans, such as skin lesions (dyspigmentation, keratosis), peripheral vascular diseases, reproductive toxicity, and neurological effects (Abernathy et al. 1999).

Exposure to arsenic typically results from either oral arsenic consumption through contaminated drinking water, soil, and food, or arsenic inhalation in an industrial work setting. Arsenic-contaminated drinking water has been associated with increased mortality of bladder and lung cancer in Chile (Marshall et al. 2007) and with increased mortality of both noncancerous causes and cancers in Bangladesh (Sohel et al. 2009). In the human arsenic metabolic pathway, inorganic pentavalent arsenic (As^V^) is converted to trivalent arsenic (As^III^), with subsequent methylation to monomethylated and dimethylated arsenicals (MMA, DMA, respectively) (Drobna et al. 2009). The general scheme is as follows:


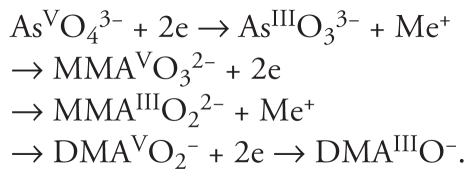


Methylated arsenicals, especially MMA^III^, are considered more toxic than inorganic As^III^ both *in vivo* (in animals) (Petrick et al. 2001) and *in vitro* (human cell lines) (Styblo et al. 2002). Several mechanisms by which arsenical compounds induce tumorigenesis have been proposed, including oxidative stress (Kitchin and Wallace 2008), genotoxic damage and chromosomal abnormalities (Moore et al. 1997a; Zhang et al. 2007a), and cocarcinogenesis with other environmental toxicants (Rossman et al. 2004); epigenetic mechanisms, in particular, have been reported to alter DNA methylation (Zhao et al. 1997).

It is generally believed that arsenic does not induce point mutations, based on negative findings in both bacterial and mammalian mutagenicity assays (Jacobson-Kram and Montalbano 1985; Jongen et al. 1985). Arsenic does induce deletion mutations, but arsenical compounds vary in their potency (Moore et al. 1997b). With respect to arsenic’s ability to induce chromosomal alterations in humans, studies in the early 1990s showed that the cell micronucleus assay could be used as a biological marker of the genotoxic effects of arsenic exposure (Smith et al. 1993). Later studies validated this assay and demonstrated higher frequencies of micronuclei in individuals who were chronically exposed to arsenicals (Moore et al. 1997a). Analysis of chromosomal alterations in DNA from bladder tumors of 123 patients who had been exposed to arsenic in drinking water showed that tumors from patients with higher estimated levels of arsenic exposure had higher levels of chromosomal instability than did tumors from patients with lower estimated levels of exposure, suggesting that bladder tumors from arsenic-exposed patients may behave more aggressively than do tumors from unexposed patients (Moore et al. 2002). Based on these overall findings, a plausible and generally accepted mechanism for arsenic carcinogenicity is the induction of structural and numerical chromosomal abnormalities through indirect effects on DNA. However, as has been demonstrated for several tumors, including urothelial and hematological malignancies (Fournier et al. 2007; Muto et al. 2000), it is likely that interrelated genetic and epigenetic mechanisms together contribute to the toxicity and carcinogenicity of arsenic (Hei and Filipic 2004; Zhao et al. 1997).

## Epigenetic Modifications Induced by Arsenic

Epigenetic alteration, which is not a genotoxic effect, leads to heritable phenomena that regulate gene expression without involving changes in the DNA sequence (Feinberg and Tycko 2004) and thus could be considered a form of potentially reversible DNA modification. Recent mechanistic studies of arsenic carcinogenesis have directly or indirectly shown the potential involvement of altered epigenetic regulation in gene expression changes induced by arsenic exposure. We recently showed that urinary defensin, beta 1 (DEFB1) protein levels were significantly decreased among men highly exposed to arsenic in studies conducted in Nevada (USA) and in Chile (Hegedus et al. 2008). DNA methylation is thought to play a role in regulating *DEFB1* expression (Sun et al. 2006). Follow-up studies are under way in our laboratory to determine if reduced levels of DEFB1 in exposed populations are due to arsenic-induced targeted gene silencing. Several studies have observed extensive changes in global gene expression in individuals after arsenic exposure (Andrew et al. 2008; Bailey et al. 2009; Bourdonnay et al. 2009; Xie et al. 2007). Further, maternal exposure to arsenic has been shown to alter expression of transcripts in the mouse fetus (Liu et al. 2008) and human newborn (Fry et al. 2007). Because epigenetic processes are major regulators of gene expression, these findings suggest that dysregulation of epigenetic processes could contribute mechanistically to arsenic-induced changes in gene expression and cancer, affecting both people exposed to arsenic directly and those of future generations in a heritable manner, without directly altering the genome. Dysregulation of epigenetic processes could also contribute to vascular disease (Yan et al. 2010) and neurological disorders (Urdinguio et al. 2009).

Many groups have directly examined the association of arsenic exposure on epigenetic phenomena; because the technologies used to study the various epigenetic modifications are developing rapidly, we believe that a review of current findings from the literature is warranted. We conducted a PubMed search (National Center for Biotechnology Information, U.S. National Library of Medicine, Bethesda, MD) through April 2010 and identified studies using variable keywords, such as “arsenic AND DNA methylation,” “arsenic AND microRNA,” “arsenic AND histone modification,” and “arsenic AND epigentics AND epigenomics.” Our goal was to include all the studies we could find, and thus the reference lists of the identified studies were also reviewed to identify other relevant studies. Although epigenetic alterations may contribute to effects of arsenic on both cancer and noncancer outcomes, in this article we summarize the recent *in vitro* and *in vivo* research findings on the potential role of arsenic-mediated epigenetic alterations in arsenic-induced toxicity and carcinogenicity. We discuss three major epigenetic mechanisms proposed to play roles in arsenic-induced carcinogenesis: altered DNA methylation, histone modification, and microRNA (miRNA) expression. We also propose future directions that can further inform our understanding of the epigenetic and overall mechanisms underlying the effects of arsenic.

## Arsenic Exposure and DNA Methylation

**Figure 1:**
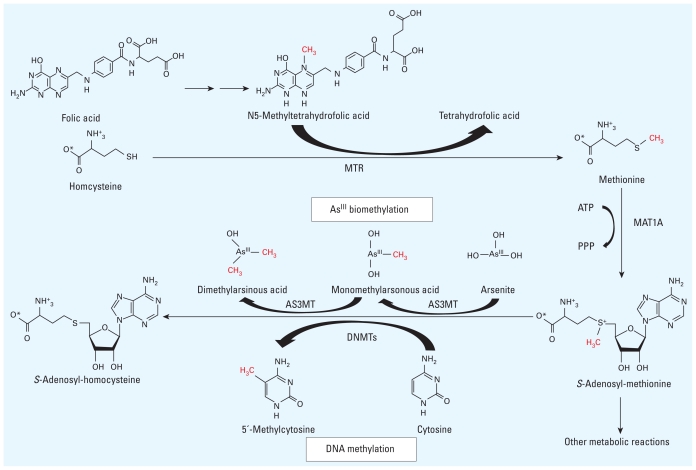
Simplified scheme of SAM synthesis and its involvement in arsenic and DNA methylation. The human arsenic metabolic pathway involves a series of methylation reactions; both arsenic metabolism and DNA methylation require SAM as the methyl donor. Here we show the intermediate steps of SAM synthesis and its involvement in the methylation of DNA and arsenic. Abbreviations: AS3MT, arsenic (+3 oxidation state) methyltransferase; ATP, Adenosine-5′-triphosphate; MAT1A, methionine adenosyltransferase I; MTR, 5-methyltetrahydrofolate-homocysteine methyltransferase; PPP, tripolyphosphate.

DNA methylation is tightly regulated in mammalian development and is essential for maintaining the normal functioning of the adult organism (Schaefer et al. 2007). Altered DNA methylation has been associated with several human diseases (Robertson 2005). Global genomic DNA hypomethylation is a hallmark of many types of cancers (Esteller et al. 2001), resulting in illegitimate recombination events and causing transcriptional deregulation of affected genes (Robertson 2005). In mammalian systems, DNA methylation occurs predominantly in cytosine-rich gene regions, known as CpG islands, and serves to regulate gene expression and maintain genome stability (Yoder et al. 1997). DNA methyltransferases (DNMTs) are responsible for transferring a methyl group from the *S*-adenosyl methionine (SAM) cofactor to the cytosine nucleotide, producing 5′-methylcytosine and *S*-adenosyl homocysteine (Figure 1) (Razin and Riggs 1980). Three different families of *DNMT* genes have been identified so far: *DNMT1*, *DNMT2*, and *DNMT3* (Robertson and Wolffe 2000).

### Mechanisms of arsenic-induced changes in DNA methylation

An association between arsenic-induced carcinogenesis and DNA methylation was proposed because arsenic methylation and DNA methylation both use the same methyl donor, SAM (Figure 1). SAM is a coenzyme involved in > 40 metabolic reactions that require methyl group transfers (Chiang et al. 1996; Loenen 2006; Reichard et al. 2007). Because SAM is the unique methyl group donor in each conversion step of biomethylation of arsenic, long-term exposure to arsenic may lead to SAM insufficiency and global DNA hypomethylation (Coppin et al. 2008; Goering et al. 1999; Zhao et al. 1997). Further, because SAM synthesis requires methionine, an essential amino acid in humans, dietary methyl insufficiency could exacerbate effects of arsenic on DNA methylation (Figure 1) (McCabe and Caudill 2005). Indeed, human exposure to arsenic often occurs in relatively resource-poor populations in developing countries that also may have low dietary intakes of methionine (Anetor et al. 2007). In addition to its effect on SAM availability, arsenic can directly interact with DNMTs and inhibit their activities. Several studies have shown that arsenic exposure leads to a dose-dependent reduction of mRNA levels and activity of DNMTs both *in vitro* and *in vivo*, including *DNMT1*, *DNMT3A*, and *DNMT3B* (Ahlborn et al. 2008; Cui et al. 2006b; Fu et al. 2007; Reichard et al. 2007).

### Arsenic and global DNA hypomethylation

Global DNA hypomethylation is expected to result from arsenic exposure through both SAM insufficiency and reduction of *DNMT* gene expression (Reichard et al. 2007). Arsenic exposure has been reported to induce DNA hypomethylation *in vitro* and in animal studies (Table 1). For example, rats (Uthus and Davis 2005) and mice (Chen et al. 2004; Okoji et al. 2002; Xie et al. 2004) exposed to As^III^ for several weeks displayed global hepatic DNA hypomethylation. Similarly, exposure of fish to As^III^ for 1, 4, or 7 days resulted in sustained DNA hypomethylation compared with nonexposed fish (Bagnyukova et al. 2007). Studies in cell lines *in vitro* yielded similar results, with a reduction in global genomic DNA methylation resulting from As^III^ exposure (Table 1) (Benbrahim-Tallaa et al. 2005; Coppin et al. 2008; Reichard et al. 2007; Sciandrello et al. 2004; Zhao et al. 1997). In contrast to the animal and *in vitro* findings, there are limited human population studies available. A cross-sectional study of 64 people reported by Majumdar et al. (2010) indicated that exposure to arsenic-contaminated water (250–500 μg/L) was associated with global DNA hypermethylation. However, the participants in the highest estimated exposure group (> 500 μg/L) had methylation levels that were comparable with those in the two lowest groups. The one possible reason for this inconsistency may be that the actual intake of arsenic into the body is different in the participants whose exposures were estimated based on the concentrations in their drinking water. In another well-designed nested case–control study, Pilsner et al. (2007) assessed the relationship between arsenic and DNA methylation in 294 participants and observed a positive association between urinary arsenic and DNA hypermethylation. Plasma folate level apparently has a significant effect on the level of DNA methylation because a dose–response relation was evident only among participants with adequate folate levels (≥ 9 nmol/L) when estimates were stratified according to plasma folate level after controling for other factors. In a separate but closely related nested case–control study, Pilsner et al. (2009) found that individuals with hypomethylation of peripheral blood leukocyte (PBL) DNA were 1.8 (95% confidence interval, 1.2–2.8) times more likely to have skin lesions 2 years later after adjusting for age, urinary arsenic, and other factors. Pilsner et al. (2009) speculated that

Adequate folate may be permissive for an adaptive increase in genomic methylation of PBL DNA associated with [arsenic] exposure, and that individuals who are similarly exposed but in whom the increase in genomic DNA methylation does not occur (or cannot be sustained) are at elevated risk for skin lesions.

Further studies are required to determine if exposure to As^III^ has differential effects on the status of DNA methylation across tissues, cells, and species.

### Arsenic and gene promoter methylation

Although the effects of arsenic exposure on global genomic DNA methylation remain unclear, DNA hypomethylation or hypermethylation of promoters of some genes has been reported in human skin cancer (Chanda et al. 2006) and bladder cancer (Chen et al. 2007; Marsit et al. 2006c) associated with arsenic exposure. It has also been observed in human cell lines (Chai et al. 2007; Fu and Shen 2005; Jensen et al. 2008; Mass and Wang 1997), animal cell lines (Chen et al. 2001, Takahashi et al. 2002), animals (Cui et al. 2006a; Okoji et al. 2002; Waalkes et al. 2004), and humans (Chanda et al. 2006; Chen et al. 2007; Marsit et al. 2006b; Zhang et al. 2007b) exposed to arsenic (Table 2). Although this gene-specific effect observed in these studies could be due to study bias, because researchers examined only a small group of genes, the similar methylation pattern repeatedly reported in the same genes after arsenic exposure might also suggest that arsenic could selectively target specific genes. However, little is known about how DNA methylation is targeted to specific regions (Jones and Baylin 2002). Hypo- and hypermethylation of genes could mediate carcinogenesis through up-regulation of oncogene expression or down-regulation of tumor suppressor genes, respectively. Both observations have been reported. Hypomethylation of the promoter region of oncogenic *Hras1* and an elevated *Hras1* mRNA level was demonstrated in mice treated with sodium arsenite (Okoji et al. 2002). Similar results on mRNA expression and promoter hypomethylation of *Hras1* and *c-myc* were also observed *in vitro* (Chen et al. 2001; Takahashi et al. 2002). The evidence has linked overexpression of *Esr1* (estrogen receptor 1) gene with estrogen-induced hepatocellular carcinoma in mice (Couse et al. 1997). Arsenic exposure leads to overexpression of the *Esr1* gene resulting from hypomethylation of its promoter region, indicating an association between overexpression of *Esr1* and arsenic hepatocarcinogenesis (Chen et al. 2004; Waalkes et al. 2004).

Dose-dependent hypermethylation at the promoter region of several tumor suppressor genes [e.g., *p15*, *p16*, *p53*, and death-associated protein kinase (*DAPK*)] was induced by arsenic exposure *in vitro* and *in vivo* (Boonchai et al. 2000; Chanda et al. 2006; Fu and Shen 2005; Mass and Wang 1997; Zhang et al. 2007b). In a population-based study of human bladder cancer in 351 patients, *RASSF1A* and *PRSS3* promoter hypermethylation was positively associated with toenail arsenic concentrations, and promoter hypermethylation in both genes also was associated with invasive (vs. noninvasive low grade) cancer (Marsit et al. 2006b). This outcome was recapitulated in arsenic-induced lung cancer in A/J mice, in which the arsenic exposure reduced the expression of *RASSF1A* resulting from hypermethylation of its promoter region and was associated with arsenic-induced lung carcinogenesis (Cui et al. 2006a). DAPK is a positive mediator of γ-interferon–induced programmed cell death and a tumor suppressor candidate. In a study of 38 patients with urothelial carcinoma, Chen et al. (2007) reported hypermethylation of *DAPK* in 13 of 17 tumors in patients living in arsenic-contaminated areas compared with 8 of 21 tumors from patients living in areas not contaminated with arsenic. This hypermethylation of *DAPK* was also observed in an *in vitro* study when immortalized human uroepithelial cells were exposed to arsenic (Chai et al. 2007). The increase of DNA hypermethylation of promoter in *p16* was observed in arseniasis patients compared with people with no history of arsenic exposure (Zhang et al 2007b). In another study Chanda et al. (2006) examined the methylation status of promoters in *p53* and *p16* in DNA extracted from peripheral lymphocytes and observed an increase of methylation in both *p53* and *p16* associated with an estimated arsenic exposure in a dose-dependent manner. However, this same study also showed that the subjects from the highest arsenic exposure group exhibited hypomethylation of both *p53* and *p16*. Chronic exposure to arsenic *in vitro* has been shown to induce malignant transformation in several human cell types (Benbrahim-Tallaa et al. 2005; Zhao et al. 1997) in which the alteration of DNA methylation level has been shown to be involved (Jensen et al. 2008, 2009a; Zhao et al. 1997).

### Summary

Arsenic does not fall into the classic model of carcinogenesis because it is not efficient at inducing point mutations or initiating and promoting the development of tumors in experimental animals. One likely mechanism by which arsenicals operate is through the disruption of normal epigenetic control at specific loci, which may result in aberrant gene expression and cancer (Andrew et al. 2008; Xie et al. 2007). Although there is increasing evidence that arsenic exposure alters methylation levels in both global DNA and promoters of some genes, the current available studies are essentially descriptive and difficult to interpret because of the complexity of the study populations and limited information provided in the reports. Studies are needed that systematically investigate DNA methylation on a genomewide level in arsenic-exposed cell lines and in target tissues, such as exfoliated bladder cells, from well-characterized arsenic-exposed human populations, or in tumor tissue from arsenic-associated cancers. Such studies would help to clarify potential effects of arsenic exposure on DNA methylation and carcinogenesis.

## Arsenic Exposure and Histone Modification

**Figure 2:**
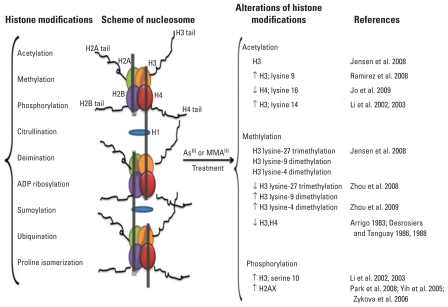
Histone modifications affected by As^III^ and MMA^III^ exposure. Major posttranscriptional histone modifications of the nucleosome are listed on the left. Modifications of specific histone proteins reported in the literature as altered by arsenic exposure are shown on the right.

Chromatin is structured within the cell nucleus in units called nucleosomes, in which DNA is packaged within the cell. The nucleosome core particle consists of stretches of DNA (~ 146 bp) wrapped in left-handed superhelical turns around a histone octamer consisting of two copies each of the core histones H2A, H2B, H3, and H4 (Luger et al. 1997). Although H1 does not make up the nucleosome “bead,” H1 plays a role in keeping in place the DNA that has wrapped around the nucleosome (Figure 2). From a structural and functional perspective, histones have different characteristics depending on the number of amino acids and the number and type of covalent modifications in these residues. These covalent modifications, found in the tails of the histone chains, influence many fundamental biological processes including acetylation, methylation, phosphorylation, citrullination, ubiquitination, sumoylation, ADP ribosylation, deimination, and proline isomerization (Kouzarides 2007) (Figure 2). To date, published studies on histone modifications and arsenic toxicity have focused on acetylation, methylation, and phosphorylation.

### Histone acetylation

Histone acetylation is a dynamic and reversible event (Glozak and Seto 2007), in which the acetylation status of lysine residues in the histone tail is regulated by two antagonistic enzyme classes, histone acetyltransferases (HATs) (Sterner and Berger 2000) and histone deacetylases (HDACs) (Cress and Seto 2000). Using acetyl coenzyme A as an acetyl group donor, HATs enzymatically transfer a single acetyl group to the ɛ-amino group of specific lysine side chains within the histone’s basic N-terminal tail region, whereas HDACs remove the acetyl group from the lysine residues.

Evidence for an association between altered histone acetylation and arsenic-induced toxicity continues to be strengthened. In the early 1980s, arsenic exposure was shown to significantly reduce histone acetylation in *Drosophila* (Arrigo 1983). More recently, changes in histone H3 acetylation have been observed in association with As^III^- and MMA^III^-induced malignant transformation of human urothelial cells *in vitro*; these modifications apparently are arsenic specific because the co-occurring changes in both As^III^- and MMA^III^-induced malignant transformation are significantly more frequent than those occurring by random chance (Jensen et al. 2008). Further, Jensen et al. (2008) reported DNA hypermethylation in a number of the hypoacetylated promoters identified in the study, suggesting that arsenic coordinately targets genes through dysregulation of different epigenetic mechanisms contributing to malignant transformation. Recently, we showed that the global level of H4K16 acetylation in human bladder epithelial cells was reduced in a dose- and time-dependent manner by both As^III^ and MMA^III^ treatment (Jo et al. 2009). Moreover, knockdown of *MYST1*, the gene responsible for H4K16 acetylation, resulted in increased cytotoxicity from arsenical exposure in human bladder epithelial cells, suggesting that H4K16 acetylation may be important for resistance to arsenic-induced toxicity.

Interestingly, As^III^ exposure has also been shown to induce elevated histone acetylation, which was reportedly responsible for the up-regulation of genes involved in apoptosis or the response to cell stress after exposure to arsenic (Li et al. 2002, 2003). This result probably is mediated by HDACs. As^III^ has been shown to inhibit *HDAC* genes that correlate with increased global histone acetylation (Ramirez et al. 2008). The level of inhibition is comparable with that of the well-known HDAC inhibitor trichostatin A (Drummond et al. 2005). Together, these studies clearly provide evidence that histone acetylation is dysregulated by arsenic exposure, but further work is needed to understand the underlying mechanisms and to clarify the net effect of altered histone acetylation on arsenic-induced toxicity and carcinogenesis.

### Histone methylation

Like acetylation, histone methylation is also a reversible process. However, unlike acetylation, which occurs only on lysine residues at the histone tail, histone methylation occurs on both lysine and arginine residues (Martin and Zhang 2005; Wysocka et al. 2006). In mammals, histone methylation is usually found on histone H3 and H4, although it also occurs on H2A or H2B. Arginine methylation is catalyzed by the enzyme arginine *N*-methyltransferase (Wysocka et al. 2006), whereas lysine methylation is catalyzed by two different classes of proteins, the SET-domain–containing protein family and the non-SET-domain proteins DOT1/DOT1L (Martin and Zhang 2005). Histone methylation can occur in the monomethyl, symmetrical dimethyl, and asymmetrical dimethyl states and in the trimethyl group states, in contrast to the single acetyl group added during acetylation (Klose and Zhang 2007). Histone methylation was considered a static modification until recent years, when enzymes were found to be capable of antagonizing histone arginine methylation or directly removing a methyl group from a lysine residue of histone (Klose and Zhang 2007). These enzymes include peptidylarginine deiminase enzymes and amine oxidase– and JmjC domain–containing histone demethylase enzymes.

Accumulating evidence implicates the aberrant loss or gain of histone methylation in tumorigenesis (Schneider et al. 2002). Arrigo (1983) first reported that exposure to arsenic in *Drosophila* cells led to a complete abolishment of methylation of histones H3 and H4, and the effect on H3 was later confirmed by other investigators (Desrosiers and Tanguay 1986, 1988). The response to arsenic exposure in the mammalian cell is more complex, and As^III^ treatment can lead to differential effects on the methylation of H3 lysine residues, including increased H3 lysine 9 dimethylation (H3K9me2) and H3 lysine 4 trimethylation (H3K4me3) and decreased H3 lysine 27 trimethylation (H3K27me3) (Zhou et al. 2008). Zhou et al. (2009) showed that 1 μM arsenite significantly increased H3K4me3 after 24-hr or 7-day exposures in human lung carcinoma A549 cells. Importantly, H3K4me3 remained elevated, apparently inherited through cell division, 7 days after the removal of arsenite. Elevated H3K9me2, mediated by increased levels of histone methyltransferase G9a protein (Zhou et al. 2008), correlates with transcriptional repression (Peterson and Laniel 2004) and has been shown to be involved in the silencing of tumor suppressers in the cancer cell lines (Esteve et al. 2007; McGarvey et al. 2006). However, data on the patterns of histone methylation induced by arsenic exposure are limited, and further studies are required to decipher the relationship between altered histone methylation and gene expression, as well as its effect on arsenic-induced carcinogenesis.

### Histone phosphorylation

All four core histone proteins, H2A, H2B, H3, and H4, and the linker histone H1 can be posttranslationally modified by phosphorylation. Cyclin-dependent kinases are believed to be responsible for H1 phosphorylation (Swank et al. 1997). Several kinases are able to phosphorylate H2A and H2B, such as ataxia telangiectasia mutated for H2AX (Burma et al. 2001). Phosphorylation of H3 has been specifically implicated in cell cycle progression and regulation of gene expression (Houben et al. 2007). Similarly, phosphorylation of histone H4 (serine 1) increases during the cell cycle and is believed to be regulated by casein kinase 2 (Barber et al. 2004).

Histone phosphorylation may also contribute to arsenic-induced carcinogenesis. Although all four core histones (H2A, H2B, H3, and H4) are targets of protein kinases (Peterson and Laniel 2004), the best-studied histone phosphorylation event is that of H2AX, a form of H2A that represents up to 25% of the total H2A pool in mammals. Zykova et al. (2006) demonstrated that arsenic trioxide induces apoptosis by up-regulation of phosphorylated H2AX and may be one of the mechanisms by which arsenic trioxide acts as an antineoplastic agent (Figure 2). Little is known about histone phosphorylation and arsenic carcinogenesis. Studies have suggested that H3 phosphorylation induced by arsenic exposure might be responsible for the up-regulation of the oncogenes c-*fos* and c-*jun* (Li et al. 2003) and induction of a protoapoptotic factor, caspase 10 (Li et al. 2002). Nickel, another important metal with epigenetic effects, has been shown to induce phosphorylation of histone 3, specifically H3S10 (serine 10) via the activation of the JNK/SAPK (c-jun N-terminal kinase/stress-activated protein kinase) pathway (Ke et al. 2008). Because arsenite exposure is known to activate JNK and p38/Mpk2 kinase by inhibition of the corresponding protein phosphatases (Cavigelli et al. 1996), phosphorylation of histone H3 via the JNK/SAPK pathway might be a common mechanism of metal-induced histone modification.

Different types of histone modifications have been shown to affect gene regulation and expression in a coordinated manner. For example, *WNT5A* gene expression is up-regulated in As^III^- and MMA^III^-induced malignant transformation in uroepithelial cells in association with the enrichment of permissive histone modifications and reduction of repressive modifications in the *WNT5A* promoter region (Jensen et al. 2009b). Two modifications of histone H3, dimethylation of H3K4 and acetylation of H3K9 and H3K14, are associated with transcriptional competency, whereas the other two modifications of histone H3, trimethylation of H3K27 and dimethylation of H3K9, are correlated with transcriptional repression (Peterson and Laniel 2004).

### Summary

Although we are still in the early stages of elucidating the association between histone modifications induced by arsenic and their effects on arsenic carcinogenicity, newly available techniques such as mass spectrometry (MS)-based histone modification analysis and genomewide sequencing offer the potential to systematically characterize the altered histone modifications induced by arsenicals and the subsequent changes in gene expression.

## Arsenic Exposure and miRNA Expression

In the past few years, several laboratories have discovered a small class of non-protein-coding RNAs, called microRNAs (miRNAs), that participate in diverse biological regulatory events and are transcribed mainly from non-protein-coding regions of the genome (Bartel 2004; He and Hannon 2004). More than 700 human miRNAs have been identified to date, as documented in the miRBase database (Release 14; miRBase 2009), and it is predicted that many more exist. Each miRNA is thought to target several hundred genes, and as many as 30% of mammalian genes are regulated by miRNA (Lewis et al. 2005). miRNAs deactivate gene expression by binding to the 3′-untranslated region of mRNA with incomplete base pairing (Wightman et al. 1993). The exact mechanisms by which expression is repressed are still under investigation but may include the inhibition of protein synthesis, the degradation of target mRNAs, and the translocation of target mRNAs into cytoplasmic processing bodies (Jackson and Standart 2007). Because of the suppressive effect of miRNA on gene expression, a reduction or elimination of miRNAs that target oncogenes could result in the inappropriate expression of those oncoproteins; for example, Johnson et al. (2005) have shown that *RAS* oncogene is regulated by the let-7 miRNA family. Conversely, the amplification or overexpression of miRNAs that have a role in regulating the expression of tumor suppressor genes could reduce the expression of such genes. A prime example of this is the observation of the miR-34 family on the p53 tumor suppressor pathway (He et al. 2007).

### Altered miRNA expression and arsenic exposure

Despite the significant progress made toward understanding the biogenesis and mechanisms of action of miRNA, much less is known about the effect of environmental exposures, especially carcinogens such as arsenic, on miRNA expression. Several studies have shown that exposure to exogenous chemicals can alter miRNA expression (Kasashima et al. 2004; Pogribny et al. 2007; Shah et al. 2007). *In vitro* exposure of cells to iron sulfate or aluminum sulfate, which generate reactive oxygen species (ROS), led to the up-regulation of a specific set of miRNAs, including miR-9, miR-125b, and miR-128 (Lukiw and Pogue 2007). ROS generation resulting from arsenic exposure is thought to play a large role in arsenic- induced carcinogenesis and toxicity (Flora et al. 2007; Hei and Filipic 2004) and could potentially alter these miRNAs in a similar manner. Marsit et al. (2006a) examined the roles that arsenic and folate deficiency play in miRNA expression; these authors found that human lymphoblast TK6 cells that had been treated with sodium arsenite and cells that had been grown in folate-deficient media over a 6-day period showed similarly altered expression of five miRNAs compared with untreated controls, suggesting a common mechanism of dysregulation. One such potential mechanism is aberrant DNA methylation occurring as a result of SAM depletion (Caudill et al. 2001; Loenen 2006), which arises under conditions of arsenic exposure and folate deficiency. However, Caudill et al. (2001) found no significant decrease in global methylation in the treated compared with the control groups, suggesting more subtle or targeted effects. The induced changes in miRNA expression were not stable and returned to baseline levels upon removal of the stress conditions, suggesting that chronic exposure may be necessary to permanently alter expression of miRNAs (Marsit et al. 2006a). Arsenic trioxide, a treatment option for acute promyelocytic leukemia (APL) (Zhou et al. 2005), induces the relocalization and degradation of the nuclear body protein promyelocytic leukemia (PML) protein, as well as the degradation of PML–retinoic acid receptor-α (PML-RARα) in APL cells (Shao et al. 1998). APL patients treated with all-*trans* retinoic acid release a group of miRNAs transcriptionally repressed by the APL-associated PML-RAR oncogene (Saumet et al. 2009), suggesting that arsenicals may produce similar effects on miRNA expression in APL patients.

### Summary

Overall, these studies show that environmental carcinogen exposures can lead to altered miRNA expression profiles, which may be associated with the process of carcinogenesis. Further studies are necessary to clarify whether chronic exposure to arsenic is capable of altering miRNA expression and what biological effects are related to the altered miRNA expression.

## Epigenomic Approach Proposed for Future Studies

Emerging evidence suggests that arsenic acts through several epigenetic mechanisms. The characterization of genomewide patterns of DNA methylation, posttranslational histone modification, and miRNA expression after arsenic exposure *in vitro* and *in vivo* represents a new frontier toward our understanding of the mechanisms of arsenic toxicity and carcinogenesis. Emerging epigenomic technologies such as chromatin immunoprecipitation (ChIP)-on-chip and ChIP sequencing (ChIP-seq), global methylation, and miRNA microarrays, as well as whole genome DNA sequencing platforms, will facilitate these efforts (Schones and Zhao 2008). ChIP-on-chip and ChIP-seq, used primarily to determine how proteins interact with DNA, have the potential to clarify how epigenetic changes, particularly histone modifications, induced by arsenic exposure regulate gene expression (Park 2009). MS offers an unbiased approach to mapping the combinations of histone modifications and requires highly sensitive and precise mass measurements; for example, the difference in mass between trimethylation and acetylation is only 36 mDa. Using liquid chromatography–MS, we identified acetylation of H4K16 as a histone modification that is significantly reduced after arsenic treatment, especially with long-term exposure (Jo et al. 2009).

With the rapid development of array and sequencing-based DNA-methylation profiling technologies, global DNA methylation profiling has clearly come of age. Because epigenetic modifications alter gene expression but not gene sequence, transcriptomics may eventually allow the characterization of the expression profiles of epigenetically labile genes. Identification of the genes dysregulated through epigenetic mechanisms by arsenic exposure will further elucidate the associated biological processes and disease states. Proteomics using both conventional “bottom-up” and newer cutting-edge “top-down” MS approaches to detect labile posttranslational modifications that are often lost in conventional MS/MS experiments will allow further clarification of the resulting phenotype. The difference between these two approaches is that the materials introduced into the mass spectrometer are either peptides generated by enzymatic cleavage of one or many proteins in the “bottom-up” approach, or intact protein ions or large protein fragments in the “top-down” approach. Integration of epigenetic, transcriptomic, and proteomic data sets generated by these techniques will facilitate a more thorough understanding of the interplay of these processes under normal conditions and during arsenic exposure. Indeed, the importance of a comprehensive understanding of the epigenome has been recognized by the scientific community and is reflected in the National Institutes of Health (NIH) Roadmap Initiative (NIH 2007) with the goal of developing comprehensive reference epigenome maps and new technologies for comprehensive epigenomic analyses.

## Conclusion and Future Directions

Although experiments in suitable model systems could complement the human studies, as discussed above, there may be differences between epigenetic effects in animals and humans and between various tissues and cell types. Thus, studies in human populations exposed to high levels of arsenic will be necessary to understand how individual differences in arsenic methylation and genetic background, as well as environmental factors such as diet and age, influence the epigenetic response to chronic arsenic exposure. Studies will also be required across various tissue and cell types to identify and validate the levels and patterns of epigenetic markers in these cells. Accessible tissues such as blood may not represent a good surrogate of target tissues such as bladder, kidney, and lung. High-resolution methylation data have shown that tissues have distinct epigenetic profiles (Christensen et al. 2009; Illingworth et al. 2008), and aging and environmental exposures may alter methylation in a tissue-specific manner (Christensen et al. 2009). Thus, epigenetic profiles from disease-relevant tissues such as exfoliated bladder cells from exposed and unexposed disease-free individuals could allow early effects to be identified. Such cells could also be analyzed from individuals with arsenic- and non–arsenic-associated cancers to identify arsenic-associated tumorigenic profiles. Rosser et al. (2009) showed that it may be possible to detect bladder cancer using gene expression signatures in exfoliated bladder urothelia. Similarly, the effects of inhaled arsenic on epigenetic profiles in bronchial airway epithelial cells could be examined in exposed and unexposed disease-free individuals and those with lung cancer, as was recently done using miRNA profiling for cigarette smoke exposure (Schembri et al. 2009).

In conclusion, a comprehensive epigenomic approach may elucidate the mechanisms of arsenic-induced carcinogenesis. Such an approach would also facilitate the discovery of biomarkers of arsenic exposure and early effects, associated diseases and disease progression, and factors that confer susceptibility.

## Figures and Tables

**Table 1:** Arsenic exposure and global DNA methylation.

Model	Arsenical	Dose	Time (weeks)	Global DNA methylation	References
Human cells					

Prostate epithelial cell line RWPE-1	As^III^	5 μM	16	Hypo	Coppin et al. 2008
Prostate epithelial cell line RWPE-1	As^III^	5 μM	29	Hypo	Benbrahim-Tallaa et al. 2005
HaCaT keratinocytes	As^III^	0.2 μM	4	Hypo	Reichard et al. 2007

Animal cells					

TRL 1215 rat liver epithelial cell line	As^III^	125–500 nM	18	Hypo	Zhao et al. 1997
V79-Cl3 Chinese hamster cells	As^III^	10 μM	8	Hypo	Sciandrello et al. 2004

Animal studies					

Goldfish	As^III^	200 μM	1	Hypo	Bagnyukova et al. 2007
Fisher 344 rat	As^III^	50 μg/g body weight	12	Hypo	Uthus and Davis 2005
129/SvJ mice	As^III^	45 ppm	49	Hypo	Chen et al. 2004
C3H mice	As^III^	85 ppm	1.5	Hypo	Waalkes et al. 2004
C57BL/6J mice	As^III^	2.6–14.6 μg/g body weight	18.5	Hypo	Okoji et al. 2002
Homozygous Tg.AC mice	As^III^	150 ppm	17	Hypo	Xie et al. 2004
	As^V^	200 ppm			
	MMA^V^	1,500 ppm			
	DMA^V^	1,200 ppm			

Human subjects					

	As^III^	2–250 μg/L	NA	Hyper	Pilsner et al. 2007; Majumdar et al. 2010
	As^III^	2–250 μg/L	NA	Hypo (in skin lesion patients)	Pilsner et al. 2009

Abbreviations: Hyper, hypermethylated; Hypo, hypomethylated; NA, not available. See text for additional information on human subjects.

**Table 2:** Arsenic exposure and gene-specific (promoter) methylation status.

				Genes		
Mode	Arsenical	Dose	Time (weeks)	Hyper	Hypo	Reference
Human cells						

UROtsa urothelial cells	As^III^	1 μM	9	*DBC1*, *FAM83A*, *ZSCAN12*, *C1QTNF6*		Jensen et al. 2008
	MMA^III^	50 nM				
Uroepithelial SV-HUC-1 cells	As^III^	2, 4, 10 μM	24 or 52	*DAPK*		Chai et al. 2007
Myeloma cell line U266	As^III^	1, 2 μM	0.4	*P16*		Fu and Shen 2005
Lung adenocarcinoma A549 cells	As^III^	0.08–2 μM	0.3	*P53*		Mass and Wang 1997
	As^V^	30–300 μM	0.3			

Animal cells						

Syrian hamster embryo cells	As^III^	3–10 μM	0.3		c-*myc*, c-*Ha-ras*	Takahashi et al. 2002
	As^V^	50–150 μM	0.3			
TRL 1215 rat liver epithelial cells	As^III^	125–500 nM	8 or 18		c-*myc*	Chen et al. 2001

Animal studies						

C57BL/6J mice	As^III^	2.6–14.6 μg/g body weight	18.5		c-*Ha-ras*	Okoji et al. 2002
A/J mice	As^V^	100 ppm	74	*p16*, *RASSF1*		Cui et al. 2006a
C3H mice	As^III^	85 ppm	1.4		*ER*α	Waalkes et al. 2004

Human subjects						

	As^III^	NA	NA	*DAPK*		Chen et al. 2007
	As^III^	Variable^a^	NA	*p53*, *P16*		Chanda et al. 2006
	As^III^	NA	NA	*p16*		Zhang et al. 2007b
	As^III^	Variable^b^	NA	*RASSF1A*, *PRSS3*		Marsit et al. 2006b

Abbreviations: ERα, estrogen receptor α; Hyper, hypermethylated; Hypo, hypomethylated; NA, not available.

a Study subjects were grouped based on historical arsenic concentration in drinking water, and the range of arsenic concentration in drinking water was < 50 μg/L to > 300 μg/L.

b The estimated toenail arsenic concentration of study subjects was < 0.01 μg/L to > 50 μg/L.
